# SOMA: a preventive health governance framework for adaptive, consumer-facing digital health guidance

**DOI:** 10.3389/fdgth.2026.1902578

**Published:** 2026-09-22

**Authors:** Michael Robert Mueller

**Affiliations:** Bratislava University of Economics and Management (BUEM), Bratislava, Slovakia

**Keywords:** adaptive capacity, adaptive preventive guidance, consumer-facing digital health, digital health twin, evidence-informed digital health, health AI safety, health self-governance, preventive health governance

## Abstract

This paper introduces SOMA, a conceptual preventive health governance framework for adaptive, consumer-facing digital health guidance, designed to support everyday preventive health self-governance. Non-communicable and lifestyle-sensitive chronic diseases account for the majority of global mortality, yet existing consumer-facing digital health tools rarely provide coherent, governed preventive guidance that integrates multimodal personal health data, scientific evidence, user-defined goals, real-world feasibility, relational support, and explicit safety constraints within a unified architecture. SOMA conceptualizes preventive health self-governance as the iterative, user-governed process through which individuals interpret their evolving health state, define goals and feasibility constraints, receive evidence-informed guidance, and act within boundaries that reduce the risk of unsafe or inappropriate digital health guidance. The framework theorizes an architecture of five interdependent components: a Digital Twin Core synthesizing a multimodal Health State Vector across biological, wearable, subjective, behavioral, and contextual layers; a Scientific Intelligence Core evaluating evidence quality, uncertainty, and applicability; a Relational Avatar Core supporting disclosure, trust calibration, and user-facing communication; a Personal Health Governance Profile embedding user-defined goals, feasibility constraints, and autonomy preferences; and a Continuous Learning Engine updating the model based on outcomes, evidence, and user feedback, all bounded by non-negotiable Safety and Medical Escalation Boundaries. The theoretical contribution lies in integrating previously siloed approaches (digital health twin logic, evidence-weighted scientific intelligence, relational guidance, behavioral self-governance theory, and personal health governance) into a unified preventive architecture whose governance properties emerge from the structured interdependence of its components. Five testable hypotheses are derived from the architecture to guide a staged empirical validation programme. SOMA is a conceptual framework that has not been empirically validated and makes no claims regarding clinical effectiveness, disease prevention, or deployment readiness.

## 1 Introduction

Non-communicable diseases, including cardiovascular disease, type 2 diabetes, metabolic syndrome, and obesity-related conditions, collectively account for approximately 74 per cent of global mortality, with a substantial proportion attributable to modifiable behavioral and lifestyle risk factors (1). A defining characteristic of lifestyle-sensitive chronic disease is its trajectory: biological, metabolic, and psychosocial processes underlying these conditions develop over years or decades before reaching clinical thresholds. Prevention is therefore decided not primarily in clinical settings but through the accumulation of daily behavioral and lifestyle decisions that either sustain or erode long-term health trajectories.

Contemporary healthcare systems are organized predominantly around episodic encounters and acute problem resolution. While evidence-based preventive medicine has made substantial advances at the population level, the continuous, personalized preventive guidance that lifestyle-sensitive disease trajectories require has remained largely outside the structural capacity of routine clinical practice (2). The resulting gap is not a failure of clinical expertise but a structural mismatch between the episodic logic of healthcare delivery and the longitudinal, everyday nature of prevention.

The proliferation of consumer-facing digital health technologies, including wearable biosensors, smartphone health applications, digital coaching platforms, and AI-powered wellness tools, has expanded access to health data and enabled forms of longitudinal self-tracking previously unavailable to non-specialist users (3). A recent computational review of 6,328 papers on AI-enabled digital health promotion identified three principal intervention contexts (consumer applications, clinical preventive care, and population-level interventions) while documenting that these remain structurally fragmented and weakly governed (4). Proactive mHealth architectures based on just-in-time adaptive interventions have demonstrated the technical feasibility of continuous, sensor-driven preventive decision support (5), yet the promise of consumer-facing digital health remains unevenly realized: many tools are weakly validated, difficult for stakeholders to evaluate, and insufficiently transparent about safety, evidence quality, privacy, or governance requirements (6–8). Individuals may thus accumulate health data without the interpretive governance structure needed to act on it meaningfully, safely, and in ways genuinely aligned with their own priorities.

Consider a person in their mid-forties with elevated fasting glucose, disrupted sleep, and a sedentary work routine, none yet at a clinical threshold. Such a person may use a fitness tracker, a nutrition application, and a general-purpose AI assistant. None provides governed preventive guidance: evidence-weighted interpretation of whether the pattern is meaningful, user-governed priority-setting about which risks to address first, relational communication that acknowledges uncertainty without overstating authority, or a principled boundary specifying when professional evaluation is required. The governance problem is not the absence of data but the absence of an architecture for converting data into responsible, bounded, person-centred preventive guidance.

I argue that this governance gap reflects the absence of a theoretical framework that specifies what consumer-facing preventive digital health guidance should integrate and how its components must relate to one another. A person seeking to address emerging cardiometabolic risk must, in practice, navigate multiple uncoordinated tools (a sleep tracker, a nutrition log, a step counter, perhaps a general-purpose AI assistant), none of which provides governed, evidence-informed, safety-bounded, and person-centred preventive guidance. Recent Frontiers in Digital Health work has framed AI-supported preventive medicine as inseparable from patient autonomy, privacy, equity, and ethical governance (8), reinforcing the structural nature of this gap.

This paper introduces SOMA, a conceptual preventive health governance framework intended to address this gap. Preventive health self-governance, as SOMA theorizes it, denotes the iterative, user-governed process through which individuals interpret their evolving health state, define their goals and feasibility constraints, receive evidence-informed guidance, and act within explicit boundaries that reduce the risk of unsafe or inappropriate digital health guidance. This definition is distinct from related constructs: it is not health self-management (which presupposes an established condition), not shared decision-making (which presupposes a clinical encounter), and not health behavior change (which focuses on the outcome rather than the governance process). SOMA is explicitly not a clinical decision support system, a diagnostic tool, an acute triage interface, or a substitute for professional clinical judgment.

To support this process, SOMA proposes an integrated architecture comprising five interdependent components. A Digital Twin Core synthesizes a multimodal Health State Vector distinguishing relatively stable biological traits, dynamic health states, and contextual conditions. A Scientific Intelligence Core evaluates evidence quality, uncertainty, and applicability. A Relational Avatar Core provides user-facing disclosure support, trust calibration, and communication of guidance and uncertainty. A Personal Health Governance Profile embeds user-defined goals, risk preferences, and feasibility constraints. A Continuous Learning Engine integrates new data, outcomes, and evidence updates under safeguards against unsafe adaptation. The framework is bounded throughout by explicit Safety and Medical Escalation Boundaries.

The paper makes three contributions. First, it develops an architectural necessity argument: governed preventive self-guidance is a system property that does not follow automatically from any partial combination of the framework's components, because each partial architecture produces a specific and predictable governance failure (Section 3.1). Existing approaches, including health digital twins, just-in-time adaptive intervention systems, learning health systems (9, 10), virtual health assistants, value-sensitive design frameworks (11, 12), and trustworthy AI governance frameworks, address individual dimensions of this problem without theorizing the interdependence required to produce governed guidance. SOMA's novelty lies in specifying this interdependence, not in proposing new individual components. Second, it reframes consumer-facing health AI from isolated recommendation generation toward governed preventive self-guidance structurally bounded by feasibility, safety, and user autonomy. Third, it derives five testable hypotheses from the framework's architecture (Section 3.9) and proposes a staged empirical validation pathway grounded in the framework's own design commitments.

SOMA is a conceptual governance framework for adaptive preventive guidance, not an optimization system, deployed tool, implementation report, or clinical decision-support system.

The paper is organized as follows. Section 2 reviews seven conceptual literature streams and synthesizes the convergent gap. Section 3 presents the SOMA architecture, its integration logic, and five testable hypotheses. Section 4 describes the temporal governance structure. Section 5 presents an illustrative scenario including an edge case. Section 6 addresses safety, ethics, autonomy, and regulatory classification. Section 7 proposes a staged validation pathway. Section 8 discusses the theoretical contribution and implications. Section 9 concludes.

## 2 Conceptual background

### 2.1 AI-enabled prevention and consumer-facing digital health

The application of artificial intelligence to health promotion and disease prevention has expanded substantially, driven by the growing availability of wearable sensors, mobile health platforms, and large-scale health data (4). AI-enabled tools have been applied to behavioral risk profiling, physical activity coaching, dietary monitoring, sleep analysis, and early symptom flagging (2, 5). Interest in extending these capabilities to everyday consumer contexts, including proactive mHealth and just-in-time adaptive intervention architectures, has grown substantially. These approaches address prediction and delivery without theorizing the governance, safety, and autonomy architecture that consumer-facing preventive guidance requires.

Yet the consumer-facing digital health landscape remains structurally fragmented. Most available tools operate in isolation, without shared data architectures, governance logic, or mechanisms for integrating individual goals, practical constraints, and safety considerations into a coherent preventive process (8). Sustained modification of lifestyle-sensitive risk trajectories requires guidance that adapts over time, remains sensitive to the individual's evolving health state and life context, and operates within explicit boundaries of safety and feasibility. No existing consumer-facing tool integrates data representation, evidence evaluation, user preference governance, and explicit safety constraints within a unified preventive architecture (13).

### 2.2 Digital health twins and personalized preventive representation

Digital twin concepts have been progressively adapted to biomedical and health contexts as dynamic, continuously updated representations of individuals built from heterogeneous data streams (14, 15). In preventive health, a digital twin logic is particularly well suited to capturing the complexity of lifestyle-sensitive disease trajectories, which unfold through the interaction of biological predispositions, fluctuating physiological states, behavioral patterns, and environmental conditions over extended time horizons (16).

Recent work has advanced the digital twin paradigm as a route to personalized and predictive care through sensing and forecasting (14), as a multi-scale, multimodal architecture requiring mathematical modeling discipline rather than sensor data aggregation alone (15), and as infrastructure whose clinical translation is constrained by governance gaps, data quality variability, and the absence of user preference integration (16). A patient-specific digital twin framework (17) supports the claim that continuous, individualized synchronization is becoming technically central to personalized healthcare. The application of digital twin logic to consumer-facing preventive self-governance remains undertheorized: existing approaches are predominantly situated in clinical or research settings and do not consistently address the governance, feasibility, and safety dimensions that everyday consumer use demands.

It is this gap in governance integration that SOMA's architecture is designed to address.

### 2.3 Evidence currency, uncertainty, and continual learning

A fundamental epistemic challenge in AI-enabled health guidance is the degradation of model validity over time. Davis et al. (18) describe how clinical prediction models undergo systematic performance decay as underlying data distributions and care practices shift. In consumer-facing preventive health, the analogous problem is broader: models may become less accurate as the individual's trajectory changes, and the scientific evidence base itself may evolve, requiring continuous updating.

Kiyasseh et al. (19, 20) demonstrate that continual learning strategies can support AI model updating under distribution shift while mitigating catastrophic forgetting. These findings converge on requirements the Scientific Intelligence Core is designed to meet: evaluating evidence quality and confidence level, making uncertainty visible rather than concealing it, and maintaining scientific currency through structured updating. The Continuous Learning Engine extends this dynamically under safeguards against drift, overfitting, and unsafe adaptation.

Communicating evidence uncertainty through a relational governance interface, and making it consequential within user-defined preferences, is precisely the design challenge that SOMA's Scientific Intelligence Core and Governance Profile are designed to address.

### 2.4 Relational agents, disclosure, and trust calibration

User engagement with AI-enabled health systems is not determined by recommendation accuracy alone. Bickmore et al. (21) provide foundational evidence that the relational quality of a health interaction (its consistency, perceived sensitivity, and capacity to support honest disclosure) is a significant determinant of continued engagement and working alliance. In preventive health, accurate subjective data on symptoms, fatigue, stress, and behavioral patterns are both pertinent to health and difficult to elicit, particularly for users managing demanding work schedules, caregiving obligations, or extended periods of elevated stress.

Virtual health assistant design features, including empathy, relational behavior, and user experience quality, are associated with better user experience outcomes (22–24). Longitudinal survival analysis of a conversational agent for chronic pain management reported a median retention of 51 days, with morning check-in features as significant retention predictors, suggesting that relational interaction design can support sustained preventive engagement in everyday consumer contexts (24). The governance implication is direct: a technically accurate recommendation delivered through a poorly calibrated interface is unlikely to be acted upon, while over-trust may prevent appropriate professional consultation. SOMA's Relational Avatar Core is proposed to address this relational dimension structurally, serving as a user-facing interface supporting disclosure, trust calibration, and uncertainty communication calibrated to the user's preferences and cognitive capacity.

What those governance and safety conditions should be, and how they must be specified for consumer-facing preventive contexts, has not been systematically theorized.

### 2.5 Autonomy, value-sensitive design, and personal governance

Consent is insufficient as a governance mechanism for health AI systems: users must have ongoing, meaningful capacity to revise preferences, contest system outputs, and constrain what the system is permitted to recommend (25). Kim et al. (26) empirically show that preferences for AI involvement in healthcare decisions vary across individuals and contexts, and that standard preference elicitation instruments do not capture this heterogeneity adequately.

A connected concern is that digitally inferred preferences should not substitute for explicit, revisable expressions of choice; health governance frameworks must account for the cognitive and motivational capacity of users to exercise genuine health autonomy under actual conditions (27). A 2026 systematic review identified lack of consideration for patient preferences as a recurrent risk in AI-supported shared decision-making (28). SOMA's Personal Health Governance Profile and Goal/Utility Layer operationalize this requirement: user-defined goals, risk tolerance, and feasibility constraints function as first-order inputs constraining recommendation logic, so that the framework makes preventive guidance accountable to what the individual values and is willing to pursue.

Making user preference structures operationally consequential (not merely elicited at consent) requires the representational and evidential infrastructure that SOMA provides.

### 2.6 Digital health literacy, equity, and feasibility

Wamala Andersson and Pisano Gonzalez (29) demonstrate that meaningful digital health inclusion requires explicit design attention to heterogeneous literacy levels, language barriers, variable digital infrastructure access, and socioeconomic constraints that shape an individual's practical capacity to benefit from AI-enabled guidance. A preventive framework inaccessible to users with limited digital confidence, irregular connectivity, language barriers, or constrained economic access to required devices will systematically exclude those populations most in need of preventive support.

Beyond access, governance burden (defined in Table 1) represents a specific design risk. SOMA's Feasibility Constraints and Cognitive Load Boundary are proposed structural responses: the framework is intended to prioritize the best feasible action given actual constraints rather than the theoretically optimal action under ideal conditions.

**Table 1:** Concise SOMA concepts and boundary conditions.

Construct	Core meaning in SOMA	Boundary/why it matters
Preventive health self-governance	User-led interpretation of an evolving health state, goals, constraints, evidence-informed guidance, and bounded preventive action.	A governance process, not clinical shared decision-making or behavior change as a simple outcome.
Consumer-facing	Everyday, non-clinical digital guidance for non-specialist users without a clinical intermediary.	Because ordinary clinical safeguards are absent, governance and equity obligations increase.
Governed preventive self-guidance	A system property produced by integrated representation, evidence, preferences, communication, and safety constraints.	Not a standalone feature; it fails when core components are implemented only partially.
Adaptive preventive guidance	Preventive suggestions updated by health state, context, individual response, and evidence currency.	Not diagnostic or therapeutic; confidence remains bounded by data quality and evidence applicability.
Health state vector	A six-layer representation distinguishing biological, clinical, wearable, subjective, behavioral, and contextual data.	Separates Trait, State, and Context to avoid conflating fixed risk factors with modifiable targets.
Scientific intelligence core	The evidence subsystem that appraises quality, uncertainty, individual applicability, and evidence currency.	Separates population-level evidence strength from individual-level applicability confidence.
Relational avatar core	The user-facing subsystem for disclosure, explanation, cognitive-load management, and trust calibration.	Not a clinical authority; must communicate limits and prevent over-reliance.
Personal health governance profile	A revisable record of goals, feasibility constraints, risk tolerance, and acceptable action boundaries.	Explicit user revision remains prior to digitally inferred preferences.
Continuous learning engine	An updating mechanism for personal data, response patterns, user feedback, and scientific evidence.	Cannot override safety boundaries or uncertainty thresholds.
Safety boundary	The non-negotiable scope within which SOMA may generate lifestyle-oriented preventive guidance.	Structurally prior to all guidance outputs and not user-configurable.
Medical escalation boundary	The boundary identifying symptoms or signal patterns that require professional evaluation.	Not subject to user override; SOMA only supports appropriate escalation.
Adaptive capacity	The ability to interpret signals, act within boundaries, and reorient a preventive trajectory over time.	Conceptually distinct from self-efficacy, patient activation, and health literacy.
Governance burden	Cognitive or practical overload caused by interaction and self-management demands.	Minimized by design and evaluated as an outcome in H5.
Governance drift	Gradual divergence between recorded goals or constraints and the user's current situation.	Addressed by the monthly strategic governance cycle.
Trust calibration	Appropriate reliance on SOMA's guidance, avoiding both over-trust and under-trust.	The target is calibrated reliance, not maximum engagement.

Full operational definitions and boundary notes are provided in Supplementary Table S1.

How guidance architectures should adapt to variable user capacity, language, and device access in consumer preventive contexts remains an open design question.

### 2.7 Behavioral theoretical foundations of preventive self-governance

A preventive health governance framework that specifies an architectural logic for guiding lifestyle action requires a behavioral theoretical foundation capable of explaining why and under what conditions structured guidance translates into sustained behavioral engagement. Three behavioral theoretical traditions are particularly germane to SOMA's design logic and strengthen its architectural justification.

Self-Determination Theory (SDT) grounds SOMA's motivational logic in the claim that sustained, internalized behavior engagement depends on the structural satisfaction of three core psychological needs (autonomy, competence, and relatedness), each of which SOMA addresses through a corresponding architectural mechanism (30). SOMA's design logic supports user autonomy by architectural specification: the Personal Health Governance Profile and Goal/Utility Layer ensure that user-defined goals, preferences, and feasibility constraints constitute first-order inputs to the recommendation logic rather than supplementary settings. The Relational Avatar Core addresses the relatedness need structurally. The Scientific Intelligence Core's explainability function addresses competence: it makes the evidential basis and uncertainty of recommendations transparent, rather than delegating health decisions to an opaque system.

The COM-B model (31) provides a complementary analytical mapping. The Health State Vector and Scientific Intelligence Core address Capability constraints; the Governance Profile and Relational Avatar address Opportunity conditions; the temporal governance structure, by scaffolding repeated low-burden engagement cycles, operates on Motivation. The two frameworks are complementary: SOMA's governance architecture addresses SDT's need dimensions and COM-B's Capability, Opportunity, and Motivation categories through the corresponding architectural components described in Section 3.

Sen's Capability Approach provides a third theoretical anchor for SOMA's governance orientation (32). On Sen's account, human wellbeing should be evaluated less by resources received or behaviors performed than by the substantive freedoms individuals can exercise in practice, given their circumstances. SOMA's emphasis on feasibility-constrained guidance, equity-aware design, and governance profiles calibrated to what the individual can realistically pursue reflects this capability orientation; the adaptive capacity construct (Section 4.5) is grounded in the notion of practically situated, iteratively developed capacity for health self-governance.

The frameworks reviewed here address behavioral theory in partial ways. Translating these insights into a unified representational, evidential, and safety infrastructure is the task this paper undertakes.

### 2.8 Synthesis: the convergent gap

The seven literature streams reviewed above (AI-enabled prevention, digital health twins, evidence currency and continual learning, relational agents, autonomy and value-sensitive design, digital health literacy, and behavioral theory) identify the necessary components of governed preventive health self-guidance: personalized longitudinal data representation, evidence-weighted and uncertainty-aware scientific intelligence, relational communication and disclosure support, user-anchored governance, explicit safety boundaries, feasibility-aware guidance calibration, and a behavioral theoretical account of how governance translates into sustained action. Each stream has developed substantial understanding of one or more of these components in isolation.

Recent Frontiers in Digital Health literature has advanced each stream relevant to SOMA, including digital twins as personalized and preventive health infrastructure (14–16), consumer-facing AI explanation and safety (8), shared decision-making and preference expression (27), and digital health literacy and equitable inclusion (29). The convergent gap is not the absence of these literatures, but the lack of an account of how their functions must operate together in consumer-facing preventive digital health. Digital twin work explains representation, continual learning research explains model updating, relational-agent research explains interaction, governance and autonomy research explains revisable preference structures, feasibility and equity research explains access constraints, and behavioral theory explains motivational and capability conditions. SOMA addresses the unresolved integration problem by specifying these functions as an interdependent governed architecture rather than as separable design elements (33).

SOMA theorizes this integration. Each of the seven literature streams reviewed above advances one or more components of governed preventive self-guidance (personalized health representation, evidence-weighted intelligence, relational communication, user-anchored governance, safety boundaries, feasibility calibration, and behavioral theoretical grounding) yet, taken separately, they do not yet fully specify how these components must operate as a structurally interdependent architecture in consumer-facing preventive contexts. The digital twin and scientific intelligence literatures develop their respective domains without integrating user governance and safety boundaries into a unified system. Governance and autonomy research establishes that preference structures must be revisable but does not address the representational and evidential infrastructure that makes preferences operationally consequential for preventive guidance. It is this structured interdependence (not any individual component) that SOMA contributes.

## 3 The SOMA preventive health governance framework

### 3.1 The necessity of integration: failure modes under partial architecture

Consumer-facing digital health tools (wearable devices, nutrition applications, AI assistants, and coaching platforms) each address one dimension of preventive health without a governing architecture connecting data interpretation, evidence quality, user goals, relational support, and safety constraints into a coherent preventive process. SOMA proposes that this limitation cannot be resolved by incrementally improving individual tools. The problem is the structural absence of integration.

The argument for architectural necessity rests on the failure modes that partial implementation risks. Five dependency failures characterize partial preventive architectures: (1) health state representation without evidence evaluation and user governance risks reducing preventive AI to instrumented surveillance or weakly personalized feedback unsupported by scientific warrant; (2) evidence-weighted intelligence without user governance and relational infrastructure risks generating guidance that is epistemically calibrated yet contextually unusable (accurate in population terms but unresponsive to the individual's goals, feasibility, and life context); (3) relational communication without a safety boundary risks cultivating user trust in a system operating without principled limits on what it may recommend or when professional evaluation is required; (4) user governance preferences without continuous learning and evidence currency risk becoming static as the individual's health state evolves and the scientific evidence base changes over time; (5) continuous learning without safety constraints and uncertainty controls risks adaptation that diverges from evidence-informed norms, optimizing observed engagement signals over governed preventive outcomes.

These are not incidental implementation risks. They are predictable structural consequences of consumer-facing preventive guidance systems operating without their full complement of interdependent components. Governed preventive self-guidance is a system property that depends on the structured interdependence of its components, not one that follows automatically from isolated component additions.

### 3.2 Digital twin core and health state vector

At the representational foundation of SOMA is the Digital Twin Core, which instantiates the Adaptive Health Twin: a personalized, continuously updated representation of the individual's health across multiple data dimensions, organized around the Health State Vector.

The Health State Vector integrates data across six layers. The Biological Substrate Layer captures relatively stable biological factors: genetic predispositions, epigenetic markers, microbiome composition, baseline metabolic parameters, and indicators of systemic inflammation. The Longitudinal Clinical Layer incorporates medical history, prior diagnoses, laboratory reference values, and clinically validated health information where available. The Wearable Biosignal Layer integrates continuous data from consumer-grade sensors (heart rate variability, sleep architecture, physical activity, oxygen saturation, continuous glucose dynamics), providing high-frequency signals that may inform daily interpretation of physiological state. The Subjective, Cognitive, and Psychological Layer captures patient-reported data on symptoms, energy, stress, mood, pain, and fatigue. The Behavioral and Lifestyle Layer represents nutritional quality and timing, movement patterns, sleep routines, and the consistency of health-promoting habits. The Environmental, Exposome, and Life-Context Layer situates the individual within broader conditions of living: occupational demands, social relationships, family structure, life phase, available resources, and environmental exposures.

The six-layer structure of the Health State Vector is theoretically grounded in an ecological systems perspective on health: health states cannot be adequately represented through biological or behavioral variables alone; the individual's environmental context, occupational conditions, and social support structures are constitutive rather than merely moderating factors. The Biological Substrate and Longitudinal Clinical layers capture relatively stable and institutionally documented health characteristics; the Wearable Biosignal and Subjective/Psychological layers capture dynamic proximal state signals; and the Behavioral/Lifestyle and Environmental/Life-Context layers capture the individual's behavioral patterns and embedding conditions within their immediate and broader ecological systems. This stratification is supported by recent precision health frameworks that recognize the multidimensional nature of individual health representation, including continuous monitoring, risk stratification, and adaptive decision support, as a precondition for valid adaptive guidance (34).

A key theoretical contribution of this structure is the explicit distinction between State, Trait, and Context. Traits are relatively stable attributes (genomic characteristics, long-term metabolic baseline) that modulate interpretation but are not primary modifiable targets of short-term guidance. States are dynamic and time-sensitive signals (sleep quality, HRV, glycemic variability) that respond to behavioral inputs and represent a central domain of adaptive preventive guidance. Contextual conditions (environmental demands, social support, resource availability) determine whether a theoretically appropriate recommendation is practically actionable at a given moment.

### 3.3 Scientific intelligence core and evidence-weighted interventions

The Scientific Intelligence Core constitutes SOMA's epistemic subsystem. Its function is to evaluate the quality, relevance, uncertainty, and applicability of scientific evidence to the specific health profile and goals of a given user, and to translate this evaluation into evidence-weighted preventive guidance calibrated to confidence and appropriate caution.

Central to this subsystem is evidence currency: ongoing accountability of recommendations to the current state of scientific knowledge. The Scientific Intelligence Core distinguishes among levels of an evidence hierarchy (from randomized controlled evidence through prospective observational studies, mechanistic or preclinical findings, and n-of-1 individual response data) and weights recommendations accordingly. Beyond evidence strength, the subsystem evaluates data quality and uncertainty: wearable sensors, self-reported signals, and consumer-grade biomarkers vary substantially in reliability, and the framework's guidance must reflect this variability transparently. An important epistemological caveat applies to consumer lifestyle interventions: the majority of long-term behavioral evidence derives from population studies, and the applicability of population-level associations to individualized preventive recommendations requires explicit confidence adjustment. The subsystem therefore distinguishes between population-level evidence strength and individual-level applicability confidence, communicating to the user not only that a recommendation is evidence-supported in the aggregate but what is known and unknown about its applicability to their specific profile. Explainability is a structural requirement. The subsystem makes transparent what evidence supports a recommendation, how confident the inference is, and where uncertainty remains.

### 3.4 Continuous learning engine and outcome evaluation

Preventive self-governance is a dynamic process in which the same action may produce different responses at different life phases, and in which the accumulation of personal outcome data and evolving scientific evidence continuously revises the basis for guidance. The Continuous Learning Engine addresses this dynamism by updating SOMA's internal model based on four inputs: new personal health data across the Health State Vector layers; individual response patterns (the observable effects of a given action on physiological and behavioral signals; user-reported feedback on feasibility and perceived impact; and updated scientific evidence where this materially changes the evidential basis for guidance).

This design requires explicit safeguards. Continuous adaptation without constraints risks model drift, overfitting to idiosyncratic short-term patterns, and unsafe adaptation deviating from evidence-informed norms. The Continuous Learning Engine therefore operates under a principle of minimal necessary change: updating incrementally in response to consistent patterns rather than any individual observation, and maintaining the safety boundaries and uncertainty thresholds established elsewhere in the framework.

### 3.5 Relational avatar core

The Relational Avatar Core is SOMA's user-facing interaction subsystem, serving three principal functions. First, disclosure support: subjective health data requires active user input, and its accuracy depends in part on whether the interaction is perceived as safe and appropriately responsive. The avatar is designed to facilitate meaningful disclosure through interaction patterns calibrated to the user's preferences and current state. Second, trust calibration: users must neither over-trust SOMA (thereby failing to seek professional evaluation when warranted) nor under-trust it to the point of disengagement. The avatar communicates confidence levels and uncertainty transparently and is explicit about the limits of SOMA's preventive scope. Third, the Cognitive Load Boundary: guidance is presented at a level of complexity manageable for the user, prioritizing a single actionable suggestion per interaction cycle rather than an exhaustive option set.

A risk specific to relational AI architectures of SOMA's type is parasocial relationship formation: affective orientation toward an AI agent that substitutes for human relationships or professional clinical contact. Naturalistic chatbot data indicate early dropout and dependency-oriented engagement patterns in some users (35), making relationship quality a governance concern. SOMA addresses this risk through trust calibration oriented toward appropriate rather than maximal engagement, through the Medical Escalation Boundary, which keeps clinically relevant concerns outside the relational interface, and through the Governance Profile's revisability, which allows users to reduce relational intensity without losing core governance functions. Whether these safeguards prevent problematic parasocial engagement remains an empirical question for usability and longitudinal feasibility studies.

The Relational Avatar Core is throughout a communication interface for preventive self-governance, not a clinical authority, diagnostic agent, or substitute for professional healthcare judgment.

### 3.6 Personal health governance profile and goal/utility layer

The Personal Health Governance Profile operationalizes user agency within SOMA. It constitutes a revisable record of what the individual wants the framework to support, which risks and actions are within acceptable boundaries, and when escalation to professional care is required.

At its core is the Goal/Utility Layer: an explicit representation of user-defined health goals, priorities, trade-offs, and risk tolerance, which may include specific preventive targets or broader quality-of-life priorities, as well as constraints on acceptable action types. Guided by user preferences, the Goal/Utility Layer is intended to constrain the framework's outputs by the user's expressed priorities, preventing internal utility functions from overriding individual values.

Feasibility Constraints are integrated as first-order inputs alongside goals: time availability, motivational capacity, social obligations, economic access, and current cognitive bandwidth (whether someone is working rotating shifts, managing primary caregiving responsibilities, or navigating a demanding occupational phase) all determine whether a recommendation is actionable in practice. Two structural risks are addressed by design. Governance Burden (excessive configuration demand creating administrative overload) is minimized by reducing required configuration complexity. Governance Drift (the gradual divergence between recorded preferences and current values or life situation) is addressed through user-revisable governance at the monthly cycle.

### 3.7 Safety boundary and medical escalation boundary

SOMA's Safety Boundary and Medical Escalation Boundary are inviolable architectural constraints that define the limits of the framework's preventive scope. They are structurally prior to any guidance output and are not subject to user configuration.

The Safety Boundary defines the domain within which SOMA may operate: lifestyle-oriented preventive guidance on sleep, physical activity, nutritional quality and timing, stress management, recovery behavior, and health literacy support. The Medical Escalation Boundary specifies conditions under which SOMA must defer to professional medical evaluation: acute symptoms or emergency warning signs; cardiovascular, metabolic, or inflammatory signal patterns that may warrant professional medical evaluation; findings implicating diagnostic questions, treatment decisions, or medication adjustments; and contexts involving vulnerability, including pregnancy, severe psychiatric conditions, complex multimorbidity, and user-indicated need for clinical support. Under these conditions, SOMA's role is limited to supporting the user in identifying an appropriate professional resource. SOMA is not an emergency medicine system, acute triage tool, diagnostic instrument, autonomous treatment system, or medication management interface.

### 3.8 Integration logic

The failure mode analysis in Section 3.1 establishes the structural basis for SOMA's integration logic: each component depends on the others in ways that cannot be resolved by incremental addition. The interdependence among the Digital Twin Core, Scientific Intelligence Core, Relational Avatar Core, Personal Health Governance Profile, and Continuous Learning Engine is the defining property of SOMA as a governance framework, not the sophistication of any individual component. A technically more advanced digital twin does not improve governance if safety boundaries and evidence currency infrastructure are not simultaneously present. A richer governance profile does not meaningfully constrain recommendations if the Scientific Intelligence Core cannot translate stated preferences into evidence-weighted, feasibility-adjusted outputs. Governed preventive health self-guidance is a system property, and partial implementation is not a step toward full governance but a differently structured governance gap.

Preventive health self-governance, as the framework theorizes it, is a system property emerging from the structured interaction of personalized representation, evidence-weighted intelligence, relational communication, user-anchored governance, and unconditional safety constraints. Figure 1 presents the SOMA framework architecture and the principal relationships among its components. Table 1 summarizes the core constructs and boundary conditions associated with this architecture; full operational definitions are provided in Supplementary Table S1. Table 3 (Section 8.4) compares SOMA's governance properties against existing digital health approaches.

**Figure 1:**
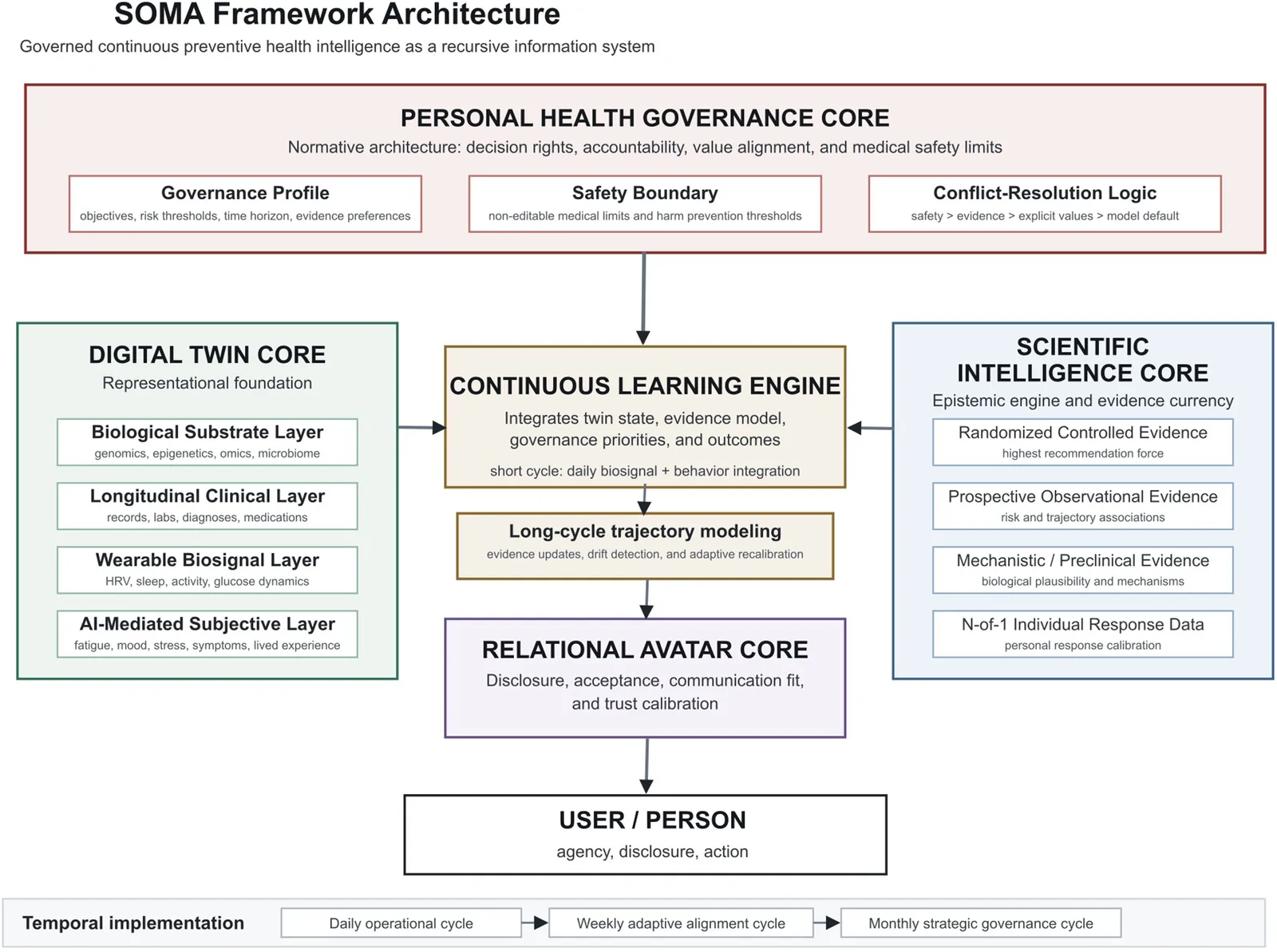
SOMA framework architecture and information flow. Information flows as a governance loop: the Digital Twin Core synthesizes the Health State Vector; the Scientific Intelligence Core produces evidence-weighted guidance candidates; the Personal Health Governance Profile constrains guidance; the Relational Avatar Core delivers user-facing guidance; and user feedback returns through the Continuous Learning Engine. Safety and Medical Escalation Boundaries are structurally prior and non-overridable. Daily, weekly, and monthly cycles operate at different resolutions. SOMA is a conceptual framework and has not been empirically validated.

Adaptive capacity as operationalized by SOMA is distinguished from three related but conceptually distinct constructs in health psychology. Self-efficacy (36) denotes a dispositional belief in one's capability to perform a specific behavior within a defined domain; adaptive capacity, by contrast, is a dynamically situated governance competence encompassing signal interpretation, boundary-aware action, and preventive trajectory reorientation across changing physiological and contextual conditions, rather than confidence in a single behavioral domain. Patient activation (37) captures knowledge, skills, and confidence for health management and healthcare system engagement, primarily operationalized as a relatively stable, cross-sectional competence level; adaptive capacity is explicitly dynamic and cultivated iteratively through SOMA's three-cycle temporal governance structure rather than assessed at a single point in time. Health literacy (38), defined as the ability to obtain, process, and understand basic health information, is a necessary but not sufficient condition for adaptive capacity: comprehension of health information does not itself entail the capacity to integrate personalized physiological signals, act within governance boundaries, and reorient a preventive trajectory as conditions change over time.

### 3.9 Testable hypotheses derived from the SOMA architecture

Consistent with the requirements of a Hypothesis and Theory article, five testable hypotheses are derived from SOMA's architectural commitments. These hypotheses are not *post hoc* additions but direct inferential consequences of the framework's design logic; they demarcate the empirical conditions under which the framework's core theoretical claims would receive support or non-support. Each hypothesis is formulated as a claim that the staged validation programme described in Section 7 is designed to test.

H1: The integration of a Personal Health Governance Profile with feasibility constraints into a preventive AI architecture will produce higher recommendation uptake rates (operationalized as self-reported action on at least one suggested guidance item per weekly cycle) compared to evidence-only preventive AI systems without personalized governance constraint logic, as evaluated in N-of-1 and controlled feasibility study designs.
H2: SOMA's Relational Avatar Core, operating with explicit trust calibration and uncertainty communication functions, will produce higher disclosure quality scores (operationalized as completeness and accuracy of subjective health layer data, assessed via interview) and lower over-trust rates (operationalized as user reports of inappropriate substitution of SOMA guidance for professional consultation) compared to standard digital health assistant interfaces without trust calibration architecture.
H3: Adaptive capacity (operationalized as the dynamically developing ability to interpret personalized health signals, act within governance boundaries, and reorient one's preventive trajectory as conditions change) will demonstrate discriminant validity from self-efficacy (36), patient activation (37), and health literacy (38) in construct validation studies using structural equation modeling or multi-trait multi-method designs.
H4: SOMA's Safety Boundary and Medical Escalation Boundary will demonstrate acceptable safety performance [defined as a false reassurance rate below 5% (proportion of escalation-warranted clinical scenarios not escalated) and an over-escalation rate below 15% (proportion of non-escalation scenarios incorrectly flagged)] in systematic simulation testing against standardized synthetic scenario libraries spanning the framework's specified escalation categories.
H5: Users engaging with SOMA's three-cycle temporal governance structure over a twelve-week longitudinal feasibility period will demonstrate a statistically and clinically meaningful reduction in governance burden (operationalized as user-reported cognitive and time costs of framework engagement, assessed at four-weekly intervals) relative to baseline, with governance burden remaining within acceptable thresholds (defined *a priori* in the study protocol) at the twelve-week endpoint.

## 4 Temporal health governance

### 4.1 Why preventive health governance requires temporal structure

Preventive health self-governance unfolds across multiple time horizons simultaneously. Not all health signals are interpretable at the same resolution, and not all governance decisions require the same frequency. Daily physiological signals carry limited information about longer-term prevention trajectories on their own, while many processes most relevant to lifestyle-sensitive chronic disease operate on lag effects that may not manifest in measurable indicators for weeks or months. Long-cycle governance questions, including whether a user's goals remain aligned with their current life situation and whether feasibility constraints remain accurate, cannot be answered at daily resolution. Cognitive load is itself a design constraint: differentiated cycles of varying depth and cognitive demand are a requirement for sustainable preventive self-governance. SOMA's temporal governance structure organizes preventive health self-governance across three conceptual cycles, each operating at a distinct time scale and serving a distinct governance function.

The theoretical justification for this structure rests on the mismatch between signal timescales and governance decisions in preventive health. Daily wearable signals are subject to instrumentation variability, acute contextual fluctuation, and biological noise unrelated to underlying health trajectories. A governance framework that treats each day's signal data as independently actionable evidence risks producing guidance that responds to short-term variation rather than to emerging patterns. The three-cycle structure is SOMA's proposed resolution: daily interactions maintain engagement and capture state signals without drawing behavioral conclusions from individual observations; weekly reviews evaluate whether pattern-level trajectories are directionally appropriate; and monthly reviews revise the foundational governance parameters with the deliberateness that strategic decisions require.

An important corollary is that the daily cycle is not an outcome monitoring or adherence enforcement mechanism. Its purpose is to support state awareness and offer a low-threshold opportunity for feasible action, not to record compliance or assess behavior against a performance target.

### 4.2 Daily operational cycle

The Daily Operational Cycle provides the user with a brief, low-cognitive-load interaction focused on recent health state signals and feasible preventive actions relevant to the day ahead. Its input domain is primarily the most recent wearable biosignal data (sleep duration and quality, heart rate variability, activity recovery status), together with brief user-reported signals on energy, mood, and perceived stress. The Relational Avatar Core facilitates this interaction, presenting guidance at a level of complexity appropriate to a brief exchange rather than a comprehensive health review. Where recent signals are ambiguous or data quality is uncertain, the daily cycle communicates this uncertainty explicitly.

The daily cycle is intended to produce a prioritized feasible action or small set of actions: a concrete, achievable lifestyle suggestion tailored to the user's current state, goals, and actual constraints for that day. This suggestion is not a directive: it is an evidence-supported option, presented with appropriate uncertainty framing, that the user is free to act upon, modify, or decline.

### 4.3 Weekly adaptive alignment cycle

The Weekly Adaptive Alignment Cycle operates at medium frequency, evaluating emerging patterns across multiple days and assessing whether the guidance being provided remains aligned with the user's trajectory, goals, and observed responses. Its input domain encompasses the full week's data across the Health State Vector, interpreted as trajectory signals rather than single-day readings. The Continuous Learning Engine provides the analytical basis for this review, identifying whether patterns are strengthening, shifting, or responding to previously suggested actions. Evidence updates applicable to the user's profile are integrated within the weekly cycle's evidence currency function.

The governance output of the weekly cycle is an adjustment: guidance is refined, reinforced, or redirected based on whether observed patterns are directionally appropriate and whether governance drift has become visible. The weekly cycle is not a compliance review; its purpose is pattern-level alignment rather than task completion.

### 4.4 Monthly strategic governance cycle

The Monthly Strategic Governance Cycle is oriented toward strategic review: the user is invited to revisit the foundational governance parameters of the framework (goals, priorities, risk tolerance, acceptable action boundaries, and life-context conditions) and to revise these as circumstances change. Life events, including changes in occupational demands, family situation, health status, or motivational capacity, may make previously recorded goals or constraints obsolete, and the monthly cycle surfaces this divergence before it results in sustained governance drift.

Risk tolerance and autonomy preference are also revisable at this cycle. A user who has increased their health literacy may wish to engage with more detailed evidence explanations; a user navigating a demanding life phase may prefer a temporarily simplified guidance mode. The monthly cadence mitigates governance burden by providing a bounded, periodic governance review.

### 4.5 Temporal integration, escalation logic, and adaptive capacity

The three cycles are interdependent layers of a single temporal governance logic. The daily cycle captures short-term state variation and supports immediate, low-cognitive-load action. The weekly cycle evaluates whether short-term patterns are translating into trajectory-level movement aligned with user goals. The monthly cycle reviews the governance parameters that frame all shorter-cycle outputs and is intended to keep the framework's foundational assumptions about user goals, constraints, and risk tolerance valid over time.

Each cycle has access to the Medical Escalation Boundary. A safety-relevant signal may arise at any time scale; medical escalation, where it occurs, is a safety output generated by the system's boundary logic, not the purpose or endpoint of temporal governance.

Over time, and subject to empirical validation, this temporal structure may support what the framework conceptualizes as adaptive capacity. Adaptive capacity requires disambiguation from related but distinct constructs in health psychology. Self-efficacy (36) denotes an individual's confidence in their ability to perform a specific behavior; it is domain-specific and does not inherently engage with the interpretive, governance, or feasibility dimensions that preventive self-governance requires. Patient activation (37) captures knowledge, skills, and confidence for managing health and interacting with the healthcare system; it is primarily operationalized as a cross-sectional measure of a relatively stable competence level rather than as a dynamically responsive governance capacity. Health literacy (38), defined as the ability to obtain, process, and understand basic health information, is a necessary but insufficient condition for adaptive capacity: comprehension of health information does not itself entail the capacity to integrate personalized signal data, translate it into feasible action, and revise one's preventive trajectory as life conditions change.

Adaptive capacity, as SOMA defines it, is the dynamically developing ability to interpret one's personalized health state signals in context, act within appropriate governance boundaries, and reorient one's preventive trajectory in response to changing physiological, behavioral, and contextual conditions over time. It is grounded in the Capability Approach (Section 2.7) rather than in a competence-measurement tradition: it is not a fixed trait to be measured but a practically situated capacity to be cultivated through the iterative governance process that SOMA's temporal structure is designed to support. Whether the framework's temporal governance structure supports the development of adaptive capacity in practice, and how it should be operationalized for empirical measurement, are questions the validation programme in Section 7 is designed to address.

## 5 Illustrative scenario

The following scenario is illustrative and intended to clarify the dependency logic of the SOMA framework; it does not report empirical data, clinical validation, or evidence of effectiveness.

### 5.1 Standard preventive governance case

Consider a 46-year-old individual with a sedentary occupation, disrupted sleep, elevated occupational stress, and recurring afternoon fatigue. Wearable data over four weeks shows reduced heart rate variability and shorter sleep duration than the individual's self-identified target. A routine blood test shows fasting glucose at the higher end of the reference range, with a modest upward trend over 18 months. The individual has no established clinical diagnosis; stated goals are cognitive performance, sleep regularity, and cardiometabolic risk management within current professional and family constraints.

Step 1: Health State Vector. SOMA organizes available data into a structured Health State Vector: the longitudinal glucose trend as a slow-moving biological signal; HRV and sleep data as dynamic signals; self-reported fatigue, stress, and sleep quality as subjective inputs; and sedentary work patterns and occupational demands as behavioral and contextual conditions. Incomplete nutritional logging is registered as epistemic uncertainty. This structured representation is the necessary precondition for Step 2; without it, the Scientific Intelligence Core cannot calibrate evidence-weighted guidance to the individual's actual health state.
Step 2: Scientific Intelligence Core. The Scientific Intelligence Core evaluates the evidence relevant to this profile (post-meal light physical activity and glycemic response, sleep regularity as a determinant of cardiometabolic risk, and brief stress regulation practices), each weighted according to the evidence hierarchy. Registered epistemic uncertainty from incomplete nutritional data is communicated to the Relational Avatar alongside the guidance outputs. Without the Governance Profile constraint applied in Step 3, this technically warranted output risks being infeasible and misaligned with the individual's actual priorities.
Step 3: Governance Profile. The Governance Profile, encoding stated goals, acceptable action types (moderate activity; not high-intensity exercise), and limited discretionary time, constrains the Scientific Intelligence Core's outputs. Time-intensive alternatives are deprioritized; the walking recommendation is anchored to an existing meal rather than added as a separate commitment. Without this constraint, technically correct guidance fails the framework's first-order requirement: that recommendations be actionable within the individual's actual circumstances.
Step 4: Relational Avatar Core. The Relational Avatar Core delivers personalized guidance in plain, uncertainty-acknowledging language calibrated to the individual's expressed preferences and cognitive load, framing recommendations as feasibility-constrained options the individual authorized the framework to prioritize. It neither overstates the authority of its outputs nor understates their evidential basis. Without the Safety Boundary defined in Step 5, this communication layer would lack the structural constraint determining the scope of what may appropriately be communicated.
Step 5: Safety Boundary. During the weekly review cycle, the Scientific Intelligence Core flags that the combination of an upward glucose trend, reduced HRV, disrupted sleep, and persistent fatigue constitutes a pattern that may warrant professional evaluation. The Safety Boundary, structurally prior to any guidance output, determines that this pattern exceeds the scope of lifestyle-oriented preventive guidance. The Relational Avatar communicates the escalation recommendation in non-alarmist language, explicitly declining to characterize the glucose reading as a clinical finding, and notes that the pattern warrants professional evaluation. This escalation is not subject to user preference override.
Step 6: Continuous Learning Engine. Following the safety escalation, the Continuous Learning Engine records the signal pattern that triggered escalation and updates confidence thresholds for similar profiles. If the individual provides a governance cycle update following professional consultation, the Governance Profile is refined accordingly. This update is constrained within the authorized guidance scope and existing safety boundaries; without evidence and safety constraints on the learning function, the framework risks optimizing for engagement rather than governed prevention.

### 5.2 Edge case: data unavailability and preference conflict

The following edge case illustrates SOMA's behavior under incomplete data and user-preference conflict; these are conditions any implementation will encounter and that the architecture must handle without compromising safety.

Consider the same individual during two weeks of intensive international travel: wearable data absent for nine of fourteen days, nutritional logging suspended, and the Governance Profile recording a preference for minimal guidance interaction during high-demand contexts.

Under substantially degraded Health State Vector coverage, the Scientific Intelligence Core registers elevated epistemic uncertainty. The daily cycle does not produce personalized recommendations for signals it does not have; instead, the Relational Avatar communicates this explicitly and offers a reduced-confidence general prompt (prioritizing sleep schedule consistency and light activity) with its uncertainty basis made transparent.

When the weekly safety cycle detects a signal pattern that, combined with the pre-existing glucose trend, would ordinarily trigger a soft safety reminder, the low-interruption preference does not override the Medical Escalation Boundary. The reminder is delivered briefly in a context-acknowledging format, without initiating a full governance review the individual has indicated they cannot complete. The monthly cycle flags the coverage gap for review upon return.

This edge case demonstrates that SOMA degrades gracefully under data unavailability (reducing guidance specificity proportionally to data quality rather than maintaining false confidence) and that user preference constraints apply within, not above, the safety architecture.

Architectural dependency conclusion. Together, the standard case and edge case demonstrate that SOMA's governed preventive health guidance is a dependency-ordered governance process: each component's output is enabled and constrained by those preceding and bounding it, such that removing any single element (structured data representation, evidence-weighted evaluation, preference governance, relational communication, a non-overridable safety boundary, or constrained adaptive learning) produces an identifiable governance failure rather than merely reduced functionality.

## 6 Safety, ethics, autonomy, and governance

Safety, ethical accountability, user autonomy, and data governance are structural requirements of a preventive health framework that determine whether any later implementation would be appropriate to evaluate or deploy. A semi-systematic review of AI governance in healthcare identified transparency and explainability as the dominant discourse themes, accounting for the majority of ethical governance attention, while patient autonomy and professional redefinition receive substantially less coverage, a distributional imbalance that underscores the need for architectures, such as SOMA, in which autonomy is a structural property rather than an afterthought (39).

### 6.1 Safety as an architectural boundary

SOMA's Safety Boundary and Medical Escalation Boundary are non-negotiable structural properties prior to any guidance output. Within the Safety Boundary, SOMA supports lifestyle-oriented preventive actions across the domains described in Section 3: sleep routines, physical activity, nutritional quality and timing, stress management, recovery behavior, and health literacy support. The Medical Escalation Boundary specifies categorical conditions under which SOMA must defer to professional medical evaluation, as enumerated in Section 3.7; these conditions are not subject to user override.

### 6.2 Consent, autonomy, and revisable preferences

User consent is necessary but insufficient. A user may consent without meaningfully expressing their goals, risk tolerance, acceptable action types, or escalation conditions. SOMA's approach to autonomy is operationalized through the Personal Health Governance Profile and Goal/Utility Layer: user-defined goals, risk tolerance settings, and acceptable action boundaries are treated as first-order constraints on the recommendation logic. The preference structure must also be revisable: the monthly governance cycle is designed to surface drift between the profile and the user's actual situation and to invite revision. Funer and Hempeler (27) caution that digitally inferred preferences should not replace explicit, revisable expressions of choice; SOMA's architecture reflects this principle.

Autonomy, in SOMA's design logic, is therefore not a property of the initial consent event but a design property of the ongoing governance relationship: it requires revisability, understandable uncertainty communication, and meaningful mechanisms for refusal or redirection at each governance cycle.

Cultural differences in health values, prevention priorities, and acceptable self-disclosure norms are a design challenge for the Governance Profile. Default settings for goal categories, acceptable action types, and risk tolerance thresholds are value-laden choices; Governance Profile design must therefore be grounded in cross-cultural user research rather than assuming culturally uniform preventive health priorities.

Harm accountability in consumer-facing preventive AI cannot be fully resolved by architectural design alone. Audit trails, documentation of guidance outputs and escalation decisions, and clear accountability structures must be integral to any implementation governance framework, not deferred as *post-hoc* compliance requirements.

### 6.3 Explainability, uncertainty, and trust calibration

SOMA's Scientific Intelligence Core is designed to make the evidential basis of guidance transparent: communicating, in accessible terms, what evidence supports a recommendation, how strong and applicable that evidence is to the individual's profile, and where uncertainty remains. Confidence levels should be proportional to actual epistemic warrant. The Relational Avatar Core is designed to support trust calibration through consistent, honest communication that acknowledges limitations, flags uncertainty, and does not present the framework as a diagnostic or clinical authority. Achieving appropriate trust calibration in practice requires empirical investigation.

Algorithmic bias represents a specific risk in the Scientific Intelligence Core and the Health State Vector. Evidence hierarchies used to weight recommendations may reflect research populations that are predominantly male, white, and from high-income health systems; applying these hierarchies to users from underrepresented groups risks producing guidance with uncertain applicability. Vulnerable users (including those with cognitive impairments, severe mental health conditions, or very limited digital literacy) may experience the Governance Profile interaction design as overwhelming, creating governance burden in precisely those users most in need of structured support. SOMA's Cognitive Load Boundary is a partial architectural response, but practical adequacy requires user research with these specific populations.

### 6.4 Privacy, data governance, and sensitive health data

SOMA integrates data across six Health State Vector layers, several of which involve categories of particular sensitivity: genomic predispositions, mental health and psychological state, and detailed life-context information. The framework's data architecture should be guided by data minimization, transparency about data processing, and user control over personal data where technically, legally, and practically feasible, including the ability to review, correct, withdraw, or delete data actively informing the Governance Profile. Privacy and data governance requirements for any implementation will need to be addressed as a dedicated component of the empirical development and validation programme.

### 6.5 Health literacy, equity, and cognitive load

Digital health literacy varies substantially across age groups, educational backgrounds, language communities, and socioeconomic contexts (38). SOMA responds through Feasibility Constraints and a Cognitive Load Boundary: guidance must remain actionable under the user's actual circumstances, and the Governance Profile must accommodate variable capacity without penalizing users who cannot sustain intensive self-monitoring. Development must also address inequities in wearables, connectivity, and health-data infrastructure, and must test whether the Scientific Intelligence Core's evidence weighting reflects populations represented in training and evaluation data. Migrant and refugee populations face interrupted records, language barriers, cross-border data governance constraints, and lower institutional trust; digital tools alone do not overcome these barriers (40). A responsible SOMA development agenda would therefore require multilingual design, reduced-data-coverage operating modes, and assessment of cross-border governance obligations.

### 6.6 Residual risks

SOMA is a conceptual framework and the following residual risks have not been resolved: inaccurate or low-quality input data generating misleading guidance; over-reliance on the framework as a substitute for professional evaluation; disengagement resulting in no preventive benefit; inequitable access to the technology and data inputs the framework requires; and governance drift between the recorded preference profile and the user's actual current values and constraints. Each represents a target for empirical investigation and iterative design refinement.

### 6.7 Implementation challenges

SOMA's architecture presupposes technical, organizational, and regulatory conditions that are not yet universally available in consumer-facing digital health. Interoperability across heterogeneous health data sources (clinical records, wearable streams, self-reported inputs) requires common data standards such as HL7 FHIR (41). Privacy regulation under GDPR constrains continuous processing of special-category health data; any SOMA implementation must assess its data flows against applicable legal bases, data minimization obligations, and cross-border transfer restrictions before deployment. Data quality and missingness are structural challenges. The Health State Vector cannot be assumed complete, and the Scientific Intelligence Core must operate under uncertainty conditions that real-world data gaps routinely create. Most fundamentally, SOMA as a technical architecture depends on complementary institutional governance (organizational policies, professional liability frameworks, clinical oversight structures, and regulatory oversight mechanisms) that no technical architecture can generate internally.

### 6.8 Regulatory classification and the conditions of responsible implementation

Any implementation of SOMA will require a dedicated regulatory assessment prior to deployment. The regulatory classification of a SOMA implementation under the EU Artificial Intelligence Act [Regulation (EU) 2024/1689] depends on implementation-specific factors that cannot be determined at the conceptual framework stage. A standard consumer-facing preventive coaching implementation is unlikely to meet the high-risk threshold under Article 6 and Annex III without a specific qualifying route, such as integration as or alongside a medical device or software classified as a medical device, or qualification under an Annex III category applicable to the implementation's context. The healthcare reference in Annex III of the AI Act concerns AI systems used by or on behalf of public authorities to evaluate eligibility for essential public assistance services, including healthcare benefits; this is a distinct context from ordinary consumer-facing preventive coaching. This classification question cannot be resolved at the conceptual framework stage and must be addressed as part of any deployment roadmap.

Regardless of risk classification, EU AI Act obligations relevant to SOMA include requirements for certain disclosures toward users who interact directly with AI systems (Article 50). For systems classified as high-risk, Article 13 of Regulation (EU) 2024/1689 additionally requires providers to ensure that sufficient information is made available to deployers to enable them to fulfil their obligations under that regulation. Further requirements include the establishment of a risk management system (Article 9) and data governance practices addressing training and operational data quality (Article 10). SOMA's architecture addresses several transparency design commitments structurally (the Scientific Intelligence Core's uncertainty communication and the Governance Profile's revisability are aligned with broader transparency design principles) though the adequacy of these architectural features for legal compliance under Article 50 or any other provision can only be assessed in a specific implementation context. Whether a specific SOMA implementation would additionally qualify as Software as a Medical Device under EU MDR is an implementation-specific question that cannot be determined at the conceptual framework stage.

The European Health Data Space [Regulation (EU) 2025/327, published 5 March 2025, in force 26 March 2025, applying from 26 March 2027, with key secondary-use and interoperability milestones in 2029 and 2031] establishes a framework for primary and secondary use of electronic health data across EU member states (42). EHDS introduces rights for individuals to access their electronic health data and to restrict its use; imposes interoperability requirements; and establishes governance structures for secondary health data use. A SOMA implementation operating across national jurisdictions or integrating data from multiple health systems would need to assess its EHDS obligations alongside those under GDPR and the EU AI Act.

## 7 Future validation pathway

### 7.1 Why empirical validation is required

SOMA specifies design requirements, proposes interdependencies, and defines a governance logic for preventive health self-governance. It does not establish whether these properties function as intended in practice, whether users engage as the temporal governance structure theorizes, or whether the guidance generated would be safe, comprehensible, acceptable, or genuinely pertinent to the health of real individuals in authentic contexts. The staged validation pathway described here is proposed as a research programme oriented toward generating this evidence incrementally. Table 2 provides a concise overview of the seven validation stages; detailed study designs, measures, sample characteristics, and evaluation criteria are provided in Supplementary Table S2.

**Table 2:** Concise SOMA staged validation roadmap.

Stage	Primary validation question	Recommended first design	Decision gate	Hypotheses
1. Expert and safety review	Is the architecture coherent and are safety boundaries complete?	Structured expert consultation; Delphi-type consensus.	Consensus threshold met; gaps resolved before user-facing work.	N/A
2. User co-design	Can users understand and configure governance preferences without excessive burden?	Participatory workshops and cognitive interviews.	Comprehensibility and governance-burden criteria met.	H1 (partial)
3. Prototype usability and cognitive-load testing	Can the avatar communicate uncertainty and safety limits without over-trust?	Mixed-method usability study.	Usability, comprehension, and over-trust criteria met.	H2, H4 (partial)
4. Safety-boundary simulation	Does escalation logic avoid false reassurance across edge cases?	Synthetic and expert-validated scenario simulation.	False-reassurance and over-escalation thresholds met.	H4
5. Longitudinal feasibility and process evaluation	Can the temporal governance cycle be sustained without increasing burden?	Single-arm 8–12 week feasibility study.	Engagement, burden, attrition, and profile-alignment criteria met.	H1, H5
6. N-of-1 and individualized response evaluation	Does guidance adapt coherently to individual response patterns?	Replicated N-of-1 designs.	Within-person consistency and recommendation-data alignment criteria met.	H3 (partial), H2 (partial)
7. Adaptive capacity construct validation	Is adaptive capacity distinct, reliable, and sensitive to change?	Scale development and validation.	Reliability, structural validity, and discriminant-validity benchmarks met.	H3

Detailed study designs, outcome measures, sample characteristics, and evaluation criteria are provided in Supplementary Table S2.

Sample sizes and formal thresholds should be specified in stage-specific protocols. The full operational roadmap is provided in Supplementary Table S2. No stage in this roadmap constitutes evidence of clinical effectiveness, disease prevention, or deployment readiness.

Empirical validation of SOMA requires clarity about which constructs require evaluation and what would constitute non-support at each stage. Constructs requiring evaluation include: the preventive health and behavioral relevance of the Health State Vector's six-layer structure and State/Trait/Context distinction; the feasibility and acceptability of the Governance Profile as a user-governable preference structure; the reliability and comprehensibility of the Scientific Intelligence Core's evidence-weighting and uncertainty communication functions; the effect of the Relational Avatar Core on disclosure quality and trust calibration; the functioning of the Safety and Medical Escalation Boundary across realistic edge-case signal profiles; and the stability of governance alignment under the temporal structure proposed in Section 4. The testable hypotheses in Section 3.9 specify the directional claims against which non-support would be interpretable.

### 7.2 Stage 1: expert and safety-boundary review

Before any user-facing prototype is developed, the framework's internal logic and safety architecture should be evaluated by independent expert reviewers drawing on perspectives from preventive medicine, digital health informatics, behavioral science, AI ethics, and clinical safety. Reviewers should evaluate conceptual coherence, the adequacy of interdependencies in Section 3, and the completeness of the escalation trigger list. Expert agreement on framework logic and safety boundaries should be treated as a necessary precondition for user-facing evaluation but does not itself constitute evidence of clinical safety.

### 7.3 Stage 2: user co-design and governance profile refinement

Participatory co-design with representative members of the target population should evaluate whether the Governance Profile structure is comprehensible, whether user-defined goals can be elicited without creating governance burden, and whether preference specification produces structures that genuinely reflect individual values. Findings from this stage should inform revisions to the governance profile before prototype usability testing proceeds.

### 7.4 Stage 3: prototype usability and cognitive-load testing

A prototype of the Relational Avatar and daily governance cycle should be evaluated for usability, comprehensibility, and cognitive load with a representative user sample. Key targets include whether users understand guidance explanations, whether uncertainty communication is comprehensible and appropriately calibrated, and whether safety escalation language is understood correctly without causing undue alarm. The prototype should be tested across digital-literacy levels. Trust-calibration process measures should be examined as process indicators. This stage addresses H2 (Relational Avatar trust calibration performance) and H4 (safety boundary performance in simulated scenarios).

### 7.5 Stage 4: safety-boundary and simulation testing

Prior to longitudinal user exposure, the framework's safety architecture should be evaluated against simulated and synthetic scenarios probing edge cases, including biomarker and signal profiles at and beyond escalation thresholds, acute symptom scenarios during a preventive interaction, diagnostic and treatment boundary scenarios, and vulnerability contexts. Two specific failure modes warrant systematic testing. False reassurance (failure to flag a signal pattern meeting escalation criteria) and over-escalation (flagging patterns within normal preventive scope) are the primary targets. H4 is evaluated at this stage: acceptable performance thresholds (false reassurance <5%, over-escalation <15%) are defined *a priori* and represent pass/fail criteria for advancing to longitudinal user testing.

### 7.6 Stage 5: longitudinal feasibility and process evaluation

Stage 5 extends the evaluation horizon to sustained use over time, addressing whether the temporal governance structure remains usable and acceptable over weeks to months. Its purpose is process evaluation: whether users engage with the three governance cycles at frequencies consistent with the framework's design assumptions, whether governance burden accumulates over time (H5), whether the Governance Profile remains aligned with users' reported values, and whether equity-relevant factors predict differential engagement. Action uptake should be measured as a process indicator, not as evidence of behavior change. H1 (governance profile uptake rates) and H5 (governance burden trajectory) are primary targets at this stage.

### 7.7 Stage 6: N-of-1 and individualized response evaluation

N-of-1 designs offer the methodologically appropriate entry point for this evaluation. These study designs involve repeated measurement of individual physiological, behavioral, and subjective signals in response to specific guidance suggestions, and are suited to evaluating whether the framework's personalization logic generates response patterns consistent with its design assumptions. Individual response data should be interpreted under explicit uncertainty quantification. The methodologically important distinction for N-of-1 evaluation is between an evidence-weighted recommendation being acted upon and the framework functioning as designed: action uptake does not confirm that the Scientific Intelligence Core's evidence weighting is valid, that the Governance Profile is accurately representing current user preferences, or that the Relational Avatar Core's trust calibration is performing as specified.

### 7.8 Stage 7: adaptive capacity construct validation

The construct of adaptive capacity (Section 4.5; H3) requires dedicated psychometric investigation before it can serve as an outcome measure in evaluative studies. Stage 7 develops and validates an adaptive capacity measure grounded in the conceptual definition in Section 4.5 and establishes its discriminant validity from self-efficacy, patient activation, and health literacy using multi-trait multi-method designs or structural equation modeling. Adaptive capacity should be measured longitudinally across the three temporal cycles to assess whether it develops in ways consistent with the framework's design logic before it is used as a primary endpoint in prospective outcome evaluation.

### 7.9 Implementation science considerations and later outcome studies

Prospective evaluation of health outcomes should be undertaken only after earlier stages have established the framework's conceptual coherence, safety-boundary adequacy, user comprehensibility, governance acceptability, longitudinal feasibility, and basic response evaluation logic. All human studies would require ethics board approval, informed consent appropriate to the sensitivity of the health data involved, and data governance reviewed against applicable regulatory requirements. Implementation science frameworks, including RE-AIM (43) and CFIR (44), provide systematic tools for evaluating reach, adoption, implementation fidelity, and maintenance at later programme stages. Regulatory evaluation of SOMA's legal classification under applicable AI and health data legislation (including the EU AI Act and, where applicable, EHDS) represents a separate and necessary component of any implementation programme that cannot be resolved at the conceptual framework stage.

## 8 Discussion

### 8.1 Theoretical contribution and synthesis

SOMA's principal contribution is the architectural necessity argument and its implication: that governed preventive health self-guidance is a system property, not a feature set. The digital health literature has developed substantial evidence for each of the framework's constituent elements (digital twin logic, evidence currency, relational agents, preference governance, continual learning, and safety boundaries) but has not theorized how these must operate together as an interdependent governance architecture in consumer-facing preventive contexts. SOMA proposes this integration and specifies the failure modes that arise under partial implementation (Section 3.1). Each missing component does not merely reduce performance; it changes what the system fundamentally is. A twin without evidence currency is instrumented surveillance; a relational interface without safety boundaries cultivates trust without principled limits; a governance profile without representational and evidential infrastructure records preferences without making them operationally consequential. Isolated component additions do not by themselves provide governed preventive self-guidance as a system property.

The integration achieves something that no partial implementation can: it makes preventive health self-governance the individual's own process. The framework's recommendation logic is constrained by what the individual values and can realistically pursue at each point in time, not merely informed by these factors. This shifts the governing criterion from population-level evidence application to an individual-level values and feasibility constraint that is structurally prior to any recommendation output. In practical terms, this means that two individuals with identical biomarker profiles and identical evidence-base classifications would receive structurally different guidance because their Governance Profiles, feasibility constraints, and temporal contexts differ. That difference is not a limitation of the framework; it is the framework's core governance property.

The reorientation this supports extends beyond SOMA's specific architecture: it establishes that consumer-facing preventive AI cannot be evaluated as accurate or effective without first establishing that its recommendations are governed, bounded by the individual's values, calibrated to the individual's capacity, and constrained by explicit safety and escalation requirements. Accuracy alone is an incomplete evaluation criterion; in the absence of governance, it may evaluate the wrong property entirely. I take this to be the paper's central methodological claim.

### 8.2 Governance, autonomy, and safety as architectural constraints

A recurring challenge in consumer-facing health AI is the treatment of safety, autonomy, and governance as supplementary features rather than as constitutive requirements that determine whether any later implementation would be appropriate to evaluate or deploy. SOMA's architecture challenges this ordering. Safety boundaries are structurally prior to any guidance output and cannot be configured away. The Goal/Utility Layer and Governance Profile are first-order constraints on the recommendation logic, not preference settings appended to a system generating population-level outputs. The Cognitive Load Boundary is a condition of the framework's validity in everyday consumer contexts.

The framework is deliberately conservative: consent, while necessary, does not suffice as a primary governance mechanism for responsible consumer-facing health AI. Explainability and uncertainty communication are not optional transparency features; they are conditions for studying and supporting calibrated reliance rather than automation bias. This governance orientation aligns SOMA with emerging scholarly discourse on autonomy-preserving AI in health (25, 45) and with the accountability-as-layered-system framing that recognizes responsibility as distributed across developers, institutions, and users rather than residing in a single governance actor (45).

### 8.3 Temporal governance as a preventive health logic

SOMA's temporal governance structure represents a further theoretical contribution distinguishing the framework from point-in-time recommendation systems. Lifestyle-sensitive chronic disease trajectories develop through the compounding of behavioral, physiological, and contextual dynamics over extended time horizons. A governance framework addressing only immediate state signals cannot meet this longitudinal challenge. The three cycles of differentiated depth and cognitive demand are the framework's proposed response, together supporting the development of adaptive capacity (defined and differentiated from self-efficacy, patient activation, and health literacy in Section 4.5) as a practically situated governance competence rather than a fixed, measurable trait.

The temporal logic is grounded in the behavioral theoretical foundations elaborated in Section 2.7: the daily cycle supports SDT's autonomy and competence needs through low-pressure, high-transparency guidance; the weekly cycle operates at the COM-B model's opportunity dimension, assessing whether contextual conditions are shifting in ways that affect the feasibility of ongoing guidance; and the monthly cycle addresses the Capability Approach's central concern: whether the framework's current parameter structure genuinely reflects the individual's actual substantive freedoms and constraints. Whether temporal governance structured in this way supports adaptive capacity in practice remains an empirical question the validation programme in Section 7 is designed to address.

### 8.4 Positioning against existing digital health approaches

SOMA's governance architecture is distinguishable from existing consumer-facing digital health approaches precisely at the points where those approaches are individually incomplete. Table 3 summarizes this comparative positioning across six governance dimensions, and Supplementary Table S3 provides the full approach-class matrix.

**Table 3:** Concise comparative governance positioning of SOMA and adjacent digital health approaches.

Governance dimension	Common limitation in adjacent approaches	SOMA's distinctive governance commitment
Personalized health state representation	Adjacent approaches often emphasize sensor-triggered delivery, clinical digital twins, session-based assistants, or encounter-based SDM rather than a persistent consumer-facing governance representation.	The six-layer Health State Vector distinguishes traits, dynamic states, and contextual conditions within a continuously updated preventive governance context.
Evidence weighting and uncertainty communication	Evidence, prediction, or decision-support logic is not consistently translated into explicit user-facing uncertainty and applicability constraints.	The Scientific Intelligence Core evaluates evidence hierarchy, uncertainty, and individual applicability before guidance is generated.
User preference as first-order constraint	User preferences are often secondary to timing, prediction, engagement optimization, or encounter-based choice.	The Personal Health Governance Profile makes goals, feasibility, and risk tolerance architectural constraints prior to recommendation logic.
Explicit safety boundary	Safety and escalation logic is often implementation-dependent, clinical-context dependent, or not standardized for consumer-facing preventive use.	The Safety Boundary and Medical Escalation Boundary are non-negotiable, prior to all outputs, and not user-overridable.
Relational and disclosure architecture	Relational agents emphasize engagement or communication, while disclosure, trust calibration, and safety constraints are not consistently integrated.	The Relational Avatar Core supports disclosure, cognitive-load management, and calibrated trust within governance boundaries.
Temporal governance and adaptive capacity	Existing approaches rarely specify a longitudinal preference-revision and adaptive-capacity logic for preventive self-governance.	The three-cycle temporal structure aligns daily action, weekly adaptation, and monthly strategic review to support adaptive capacity.

The full approach-class matrix is provided in Supplementary Table S3.

Reading guide: The table summarizes the comparative pattern across proactive mHealth/JITAI, health digital twin platforms, virtual health assistants, and SDM tools (5, 14, 15, 22, 27). It highlights typical design emphases within these approach classes, not specific commercial implementations or empirical claims that all systems within a class behave identically. The full comparison by approach class is provided in Supplementary Table S3.

### 8.5 Practical implications

SOMA's governance architecture carries implications for four stakeholder audiences.

For clinicians and clinical governance bodies, SOMA highlights that consumer-facing preventive AI operating outside clinical encounters creates a new accountability topology. Patients may present with AI-derived guidance histories that clinicians had no role in, and clinical governance bodies may need frameworks for evaluating whether consumer-facing systems their patients use have adequate safety boundaries and escalation triggers. SOMA's Medical Escalation Boundary is designed to produce structured referrals to professional evaluation, not to replace the clinical encounter.

For AI developers and digital health designers, the governance-first implication is most demanding: safety boundaries, preference structures, and escalation logic must be specified architecturally before recommendation algorithms are designed. Uncertainty communication is a design requirement; explainability that misrepresents population-level evidence as individual-level applicability is not genuine transparency. Equity must be embedded in training data requirements and Governance Profile accessibility from the earliest design stage.

For healthcare organizations evaluating consumer-facing preventive AI, the framework provides a governance property checklist: Does the system integrate personalized representation, evidence currency, relational communication, user-anchored preference governance, and explicit safety boundaries simultaneously? A system meeting only some of these criteria does not approximate SOMA's governance model; it instantiates a structurally incomplete one.

For policymakers and regulators, the governance-as-system-property argument implies that feature-level regulatory compliance may underestimate risks arising from partial implementation; the evaluative challenge is whether components function as an integrated governance architecture, not merely whether each is individually present.

### 8.6 Limitations and boundary conditions

SOMA is a conceptual framework. The architecture described has not been implemented, tested, or empirically evaluated. No safety performance has been demonstrated. The framework specifies safety boundaries as design requirements, but their adequacy under actual conditions of use has not been established. No clinical effectiveness has been demonstrated. The framework makes no claim to prevent disease, improve health outcomes, or produce any specific physiological or behavioral change.

The framework's dependency on high-quality multimodal data is a further limitation: consumer-grade devices vary substantially in reliability, and under conditions of poor data quality the guidance logic may generate poorly calibrated outputs. The graceful degradation logic described in Section 5.2 is a design requirement, not a validated behavior.

Three methodological limitations of the present paper itself warrant acknowledgment. First, the framework was developed by a single author without embedded multidisciplinary input from preventive medicine, behavioral science, clinical safety, or AI ethics. The theoretical synthesis reflects the perspectives available at that stage; Stage 1 of the validation pathway addresses this through structured expert review. Second, no user involvement was incorporated in the framework development phase; the governance structures proposed, including the Governance Profile's elicitation logic and the Relational Avatar's interaction design, have not been tested against actual user mental models, cognitive capacities, or expressed preferences. Third, the framework's conceptual assumptions implicitly reflect a high-income, digitally connected context; applicability to low-resource settings, contexts of intermittent connectivity, or populations with limited digital health literacy remains an open empirical and design question.

### 8.7 Future research

The empirical validation agenda for SOMA is described in Section 7, progressing from expert review and user co-design through prototype usability testing, safety simulation, longitudinal feasibility evaluation, N-of-1 individualized response evaluation, and adaptive capacity construct validation before outcome-oriented prospective studies. These stages also address broader questions: how governance profiles can be elicited without excessive burden; how escalation language can avoid alarm or dismissal; how individualized response data can be interpreted under uncertainty; and how temporal governance cycles can remain feasible over months of use.

## 9 Conclusion

The challenge that motivates this paper is structural rather than technological. Individuals managing lifestyle-sensitive health trajectories, before chronic disease becomes clinically manifest, lack access to a governed digital health architecture for connecting their personal health data, the available scientific evidence, their own goals and constraints, and an appropriate boundary between preventive self-governance and professional medical care. Consumer-facing digital health tools have proliferated without resolving this gap: wearable devices track signals without governing their interpretation; recommendation systems generate guidance without situating it within the individual's values, feasibility constraints, or safety limits; relational interfaces engage users without anchoring that engagement in evidence or bounded governance logic.

SOMA is proposed as a conceptual response to this gap: a digital health governance framework for continuous preventive health intelligence. It theorizes five interdependent components (a Digital Twin Core, a Scientific Intelligence Core, a Relational Avatar Core, a Personal Health Governance Profile, and a Continuous Learning Engine), bounded by non-negotiable Safety and Medical Escalation Boundaries before any guidance output. Its behavioral foundations in Self-Determination Theory, the COM-B model, and the Capability Approach connect governance to the motivational and capability conditions required for preventive self-governance. Its three temporal cycles support adaptive capacity: the situated ability to interpret health-state signals, act within appropriate boundaries, and remain oriented to a preventive trajectory over time. Five testable hypotheses (Section 3.9) anchor the validation agenda.

SOMA is a conceptual framework whose value as a scientific contribution rests on the coherence and specificity of its design logic and on the feasibility of the staged validation programme it motivates. Empirical validation of SOMA's architecture, safety boundaries, and preventive guidance logic remains the necessary next step.

## Data Availability

The original contributions presented in the study are included in the article/Supplementary Material, further inquiries can be directed to the corresponding author.
