# Supplementary material for: SOMA: a preventive health governance framework for adaptive, consumer-facing digital health guidance
Source: Front Digit Health. 2026 Sep 22;8:1902578. doi: 10.3389/fdgth.2026.1902578 (PMC13638536; doi:10.3389/fdgth.2026.1902578)
Supplement: Supplementary file 1 [file Table1.docx]

# Supplementary Tables

## Supplementary Table S1. Full operational definitions and boundary notes for key SOMA concepts.

| **Construct** | **Operational Definition** | **Safety / Boundary Note** |
| --- | --- | --- |
| Preventive health self-governance | The iterative, user-governed process through which individuals interpret their evolving health state, define goals and feasibility constraints, receive evidence-informed guidance, and act within explicit boundaries that reduce the risk of unsafe or inappropriate digital health guidance. | Distinct from health self-management (presupposes an established condition), shared decision-making (presupposes a clinical encounter), and behavior change as an outcome (rather than a governance process). |
| Consumer-facing | Denotes systems operating in everyday, non-clinical contexts for non-specialist individuals, without a clinical intermediary, professional oversight structure, or institutional governance infrastructure characteristic of medical settings. | Guidance occurs outside the ordinary safeguards of clinical encounters, heightening obligations for safety boundary specification; consumer-facing status increases rather than reduces governance and equity obligations. |
| Governed preventive self-guidance | The system property that emerges when personalized health representation, evidence-weighted intelligence, user-anchored preference governance, relational communication, and non-negotiable safety constraints operate together as an interdependent architecture; not one that follows automatically from any partial combination of the framework's components. | A system property, not a feature of any individual component; the absence of governed preventive self-guidance in consumer-facing digital health is the gap this framework addresses. |
| Adaptive preventive guidance | Evidence-weighted preventive guidance that updates in response to the individual's evolving health state, behavioral responses, life-context conditions, and evidence currency, constrained throughout by user-defined governance parameters and non-negotiable safety boundaries. | Guidance confidence is bounded by data quality and evidence applicability; not a diagnostic or therapeutic output. |
| Health State Vector | A multimodal representation of the individual's health across six data layers (biological substrate, longitudinal clinical data, wearable biosignals, subjective and psychological data, behavioral and lifestyle patterns, and environmental and life-context factors), distinguishing relatively stable Traits, dynamic States, and Contextual conditions. | Data quality variability across layers must be reflected in guidance confidence levels; the Trait/State/Context distinction reduces the risk of conflating fixed risk factors with modifiable preventive targets. |
| Scientific Intelligence Core | The epistemic subsystem evaluating the quality, uncertainty, and individual applicability of scientific evidence, translating evidence evaluations into guidance weighted by confidence level and appropriate caution, and maintaining evidence currency through structured updating. | Does not generate diagnostic conclusions, treatment recommendations, or medication guidance; population-level evidence strength is distinguished from individual-level applicability confidence. |
| Relational Avatar Core | The user-facing interaction subsystem supporting health disclosure, trust calibration, and cognitive-load management, designed to facilitate accurate subjective data elicitation and calibrated (rather than maximal) reliance on the framework's guidance. | Not a clinical authority; must communicate the limits of SOMA's preventive scope explicitly; structural safeguards against parasocial relationship formation and over-reliance are design requirements. |
| Personal Health Governance Profile (Governance Profile) | A revisable record of user-defined health goals, risk tolerance, feasibility constraints, and acceptable action boundaries, functioning as a first-order architectural constraint on all recommendation logic. | Supports ongoing user revision; digitally inferred preferences cannot substitute for explicit user expression; the monthly governance cycle is designed to surface governance drift before it accumulates. |
| Continuous Learning Engine | An adaptive updating mechanism integrating new personal health data, individual response patterns, user feedback, and scientific evidence updates, operating under a principle of minimal necessary change to maintain model validity across changing individual trajectories. | Cannot override safety boundaries or uncertainty thresholds; updates are constrained to prevent model drift, overfitting to short-term signals, and unsafe adaptation diverging from evidence-informed norms. |
| Safety Boundary | The architectural constraint defining the operational domain within which SOMA may generate preventive guidance, restricted to lifestyle-oriented actions: sleep, physical activity, nutritional quality and timing, stress management, recovery behavior, and health literacy support. | Non-negotiable; not subject to user configuration or system override; structurally prior to all guidance outputs. |
| Medical Escalation Boundary | The categorical specification of signal patterns or user-reported symptoms that exceed preventive guidance scope and require direction to professional evaluation, including acute or emergency presentations, cardiovascular and metabolic patterns warranting clinical assessment, diagnostic and treatment questions, and vulnerability contexts. | Categorical; not subject to user override; SOMA's role under these conditions is limited to supporting the user in identifying an appropriate professional resource. |
| Adaptive capacity | The dynamically developing ability to interpret one's personalized health state signals in context, act within appropriate governance boundaries, and reorient one's preventive trajectory in response to changing physiological, behavioral, and contextual conditions over time. | Empirically distinct from self-efficacy, patient activation, and health literacy (see paragraph below and Section 4.5); requires dedicated construct validation (H3; Stage 7 validation programme). |
| Governance burden | The cognitive and practical overload arising when framework-interaction or self-management requirements exceed the individual's realistic capacity to act, manifesting as administrative fatigue, disengagement, or non-completion of governance cycles. | Addressed architecturally through minimal required configuration and the monthly governance cycle's bounded review structure; operationalized as user-reported cognitive and time costs in H5. |
| Governance drift | The gradual divergence between a user's recorded governance parameters (goals, feasibility constraints, risk tolerance) and their actual current values, priorities, or life situation, typically arising without a deliberate revision event. | Addressed through the monthly strategic governance cycle, which is designed to surface drift before it results in guidance that no longer reflects the user's actual situation. |
| Trust calibration | The ongoing process by which a user develops and maintains an appropriate level of reliance on SOMA's guidance, neither substituting AI preventive guidance for warranted professional consultation (over-trust) nor disengaging from guidance of legitimate preventive value (under-trust). | Measured via over-trust rates and disclosure quality scores in H2; appropriate calibration, not maximum engagement, is the architectural target. |

**Explanation:** Supplementary Table S1 expands the concise concept overview in main-text Table 1. It functions as the operational glossary for SOMA: the second column defines each construct, and the third column clarifies the safety or boundary condition that limits how the construct should be interpreted.

## Supplementary Table S2. Full SOMA staged validation roadmap: study designs, outcome measures, sample characteristics, and evaluation criteria.

| **Stage** | **Focus** | **Study Design** | **Primary Outcome Measures** | **Sample Characteristics** | **Evaluation Criteria** | **Hypotheses Addressed** |
| --- | --- | --- | --- | --- | --- | --- |
| 1. Expert and Safety Review | Framework logic; safety-boundary completeness | Structured expert consultation; Delphi-type consensus procedure | Expert-rated adequacy of interdependency logic; completeness of escalation-trigger taxonomy; formal consensus on governance property claims | Small expert panel spanning preventive medicine, digital health informatics, behavioral science, AI ethics, and clinical safety | Pre-specified agreement threshold; identified gaps logged and resolved before Stage 2 | Not applicable |
| 2. User Co-Design | Governance Profile comprehensibility; goal elicitation without governance burden | Participatory co-design workshops; cognitive interview rounds | User-rated comprehensibility of preference elicitation; presence/absence of governance burden; alignment between elicited goals and subsequently reported priorities | Purposive sample of adults with diverse digital-literacy levels, age groups, and health contexts; equity-relevant subgroups oversampled | Pre-specified governance burden absence and goal-elicitation stability criteria | H1 (partial) |
| 3. Prototype Usability and Cognitive-Load Testing | Relational Avatar comprehensibility; uncertainty communication; safety-escalation language | Mixed-methods usability study (think-aloud, cognitive load measures, post-task interview) | System Usability Scale score; comprehension accuracy for uncertainty labels; safety-escalation message interpretation; trust calibration process measures | Purposive sample stratified by digital-literacy level, including low-literacy participants | Pre-specified usability, comprehension accuracy, and over-trust absence criteria | H2, H4 (partial) |
| 4. Safety-Boundary Simulation | Escalation-trigger performance across edge cases | Scenario simulation study using synthetic and expert-validated signal profiles | False-reassurance rate; over-escalation rate; boundary consistency across vulnerability contexts | Systematic set of synthetic profiles spanning escalation-threshold edge cases, acute symptom scenarios, and vulnerability contexts | Pre-specified false-reassurance and over-escalation thresholds defined prior to simulation | H4 |
| 5. Longitudinal Feasibility and Process Evaluation | Temporal governance structure; governance burden trajectory; equity-relevant engagement | Single-arm longitudinal feasibility study (8–12 weeks) | Governance cycle engagement rates (daily, weekly, monthly); governance burden trajectory; governance profile alignment with reported values at follow-up | Feasibility sample of adults over 8–12 weeks; include subgroup with high governance-burden risk | Pre-specified engagement, burden trajectory, and attrition criteria | H1, H5 |
| 6. N-of-1 and Individualized Response Evaluation | Scientific Intelligence Core personalization logic; response consistency | Replicated N-of-1 designs (ABA or ABAB) with individual as own control | Individual physiological and behavioral signal response to guidance; user-reported feasibility and perceived relevance; evidence-weighting consistency under explicit uncertainty quantification | Replicated N-of-1 series with sufficient washout periods | Pre-specified within-person consistency and recommendation-data alignment criteria | H3 (partial), H2 (partial) |
| 7. Adaptive Capacity Construct Validation | Psychometric validation of adaptive capacity measure | Scale development and validation study (item generation, EFA/CFA, multi-trait multi-method discriminant validity) | Internal consistency; test-retest reliability; discriminant validity from self-efficacy (36), patient activation (37), and health literacy (38); longitudinal sensitivity | Adequate psychometric sample; validation sample separate from development sample | Pre-specified psychometric benchmarks for internal consistency, structural validity, and discriminant validity | H3 |

**Explanation:** Supplementary Table S2 provides the detailed validation roadmap underlying main-text Table 2. It identifies the type of study design, outcome evidence, sample logic, and decision criteria that would be needed before later validation stages could be justified; it does not report completed studies or establish safety, effectiveness, or deployment readiness.

Notes: Sample sizes and evaluation thresholds should be formally specified in stage-specific study protocols and are not pre-determined at the conceptual framework stage. All human-participant stages require ethics board approval and informed consent appropriate to the study design.

## Supplementary Table S3. Full comparative governance matrix: SOMA and adjacent digital health approaches.

| **Governance Dimension** | **Proactive mHealth / JITAI (5)** | **Health Digital Twin Platforms (14–15)** | **Virtual Health Assistants (22)** | **SDM Tools (27)** | **SOMA** |
| --- | --- | --- | --- | --- | --- |
| Personalized health state representation | Sensor-driven, typically single-modality or narrow behavioral domain; real-time delivery optimization is the primary design focus. Multi-layer, multi-source governance integration is not a primary design specification in this approach class. | Strong: continuous, multi-source state modeling, often simulation-capable. Primarily in clinical or research contexts; consumer-facing governance architecture is less central to this class's design scope. | Session-based and user-reported data; persistent multi-layer health state modeling is not a primary design feature of this approach class. | Encounter-based; health state captured at the clinical interaction point; continuous updating is not part of the design scope. | Six-layer Health State Vector: stable traits, dynamic states, and contextual conditions distinguished. Personalized, continuously updated, consumer-facing governance context. |
| Evidence weighting and uncertainty communication | Evidence-informed behavioral algorithms; explicit uncertainty communication to users is not a standard design specification in this approach class. | Simulation and prediction models; uncertainty communication to end users is not a consistent design specification. | Responses are not consistently evidence-stratified across this approach class; uncertainty framing varies. | Evidence summaries standard in decision aids; uncertainty communicated at encounter point; not adaptive to an evolving individual profile. | Scientific Intelligence Core evaluates evidence hierarchy, applicability confidence, and uncertainty explicitly; distinguishes population-level evidence from individual applicability confidence. |
| User preference as first-order constraint | User goals supplementary to delivery timing optimization; preference structure is not typically specified as architecturally prior to recommendation logic in this approach class. | Personalization to health data; user values and feasibility constraints as first-order architectural constraints are not a primary design specification in this class. | Engagement-optimized; user values as first-order recommendation constraints are not a primary design specification. | User preference central in the shared decision-making encounter; not designed for continuity or integration with behavioral guidance logic between encounters. | Personal Health Governance Profile (goals, feasibility, risk tolerance) architecturally prior to recommendation logic; revisable across governance cycles. |
| Explicit safety boundary | Safety and escalation architecture is not a primary design specification in this approach class; scope constraints are typically implementation-dependent. | Clinical safety architecture present in medical digital twins; consumer-facing safety boundary specification is less consistently a design priority in this class. | Formal safety and escalation boundaries are not a standard design specification in this approach class. | The clinical encounter provides an implicit safety boundary; safety boundary architecture for consumer-facing continuous use is not part of the standard design scope. | Non-negotiable Safety and Medical Escalation Boundaries structurally prior to all outputs; categorical escalation trigger specification; not subject to user override. |
| Relational and disclosure architecture | Delivery-focused; formal relational and disclosure architecture are not primary design specifications in this approach class. | Data-model-focused; relational interaction design is not a primary specification in this class. | Relational engagement is the primary design focus; linkage to governance profile, evidence architecture, and safety boundary is not consistently specified. | Patient communication emphasized in the clinical encounter; relational continuity between encounters is not part of the design scope. | Relational Avatar Core: trust calibration, parasocial risk safeguards, cognitive load boundary, disclosure support, governed by and constrained within the safety and governance architecture. |
| Temporal governance and adaptive capacity | Real-time delivery orientation; multi-cycle governance logic supporting longitudinal preference revision is not a primary design specification. | Continuous state modeling; explicit longitudinal governance cycle structure is not a standard design specification in this class. | Interaction-based; cyclical governance structure is not a standard design specification. | Episodic encounter-based; longitudinal governance cycle is not part of the design scope. | Three-cycle temporal structure (daily operational, weekly adaptive alignment, monthly strategic review) designed to support adaptive capacity; subject to empirical validation. |

**Explanation:** Supplementary Table S3 expands the concise comparative positioning in main-text Table 3. Cells describe typical design emphases of adjacent approach classes rather than exhaustive claims about every implementation; the SOMA column summarizes architectural commitments that remain to be empirically validated.
